# Nutritional regulation of gut-brain immune crosstalk across the lifespan

**DOI:** 10.1080/19490976.2026.2725371

**Published:** 2026-09-04

**Authors:** Yulin Zhou, Sebastian Del Toro, Melody Y. Zeng

**Affiliations:** a Gale and Ira Drukier Institute for Children’s Health, Weill Cornell Medicine, New York, NY, United States of America; b Department of Pediatrics, Weill Cornell Medicine, New York, NY, United States of America; c Immunology and Microbial Pathogenesis Program, Weill Cornell Graduate School, New York, NY, United States of America

**Keywords:** Nutrition, gut-brain axis, metabolites, aging, neuroimmunology, microbiome

## Abstract

The gut-brain immune axis integrates microbial, immune, and neural signals to regulate neurodevelopment, homeostasis, and disease susceptibility. Early-life nutrition, particularly human milk oligosaccharides, shapes beneficial microbiota composition, enhances hippocampal plasticity, promotes anti-inflammatory microglia polarization, and fosters immune tolerance. Gut microbiota-derived metabolites, including short-chain fatty acids, tryptophan derivatives and secondary bile acids, regulate microglia maturation, astrocyte function, T cell differentiation, neurotransmitter production, and vagus nerve signaling. These processes influence synaptic pruning, neurogenesis, and neuroinflammation. Adaptive immune cells in the central nervous system, notably meningeal and infiltrating CD4 T cells, further connect peripheral immunity to neuronal responses through cytokines, such as IL-4, IFNγ, and IL-17A. Nutritional imbalances may exacerbate disease-associated microglia and pathogenic T cell activity in Multiple Sclerosis, Alzheimer’s disease, and autism spectrum disorders. In aging, diet helps mitigate “inflammaging” by countering metabolic shifts in microglia and lymphocytes. This review examines how nutrition modulates bidirectional gut-brain immune crosstalk across the lifespan.

## 1. Introduction

The gut-brain immune axis orchestrates complex interplay among the immune system, gut microbiota, and central nervous system (CNS), which profoundly shapes neurodevelopment and immune regulation, particularly during early life. This bidirectional network integrates neuronal, endocrine, and immune signals to regulate CNS homeostasis, neurogenesis, and neuroinflammation. Microglia, the resident immune cells of the CNS, are critical for synaptic pruning and supporting early neurogenesis by secreting growth factors, while adaptive immune cells, such as CD4 T cells, infiltrate the developing brain to promote microglia maturation and regulate cognitive and behavioral outcomes through cytokines, including IFNγ, IL-4 and IL-17A. The gut microbiota significantly influences this axis by producing metabolites that cross the blood-brain barrier or signal via the vagus nerve to modulate microglia and immune cell function. Early life nutrition is a key determinant of this crosstalk by shaping microbiota composition and priming immune cells. Breast milk, rich in bioactive components, particularly human milk oligosaccharides (HMOs), notably 2’-fucosyllactose, enhances hippocampal plasticity, promotes anti-inflammatory M2 microglia polarization, and fosters gut microbial diversity, which has a profound impact on neurodevelopment and immune tolerance. Dietary deficiencies or disruptions during this critical window can impair CNS immune cells and increase susceptibility to neurodevelopmental disorders. Aging also triggers shifts in microbiota and immune metabolism that contribute to neuroinflammation and neurodegeneration, underscoring the lasting impact of early nutritional status.

**Figure 1.**
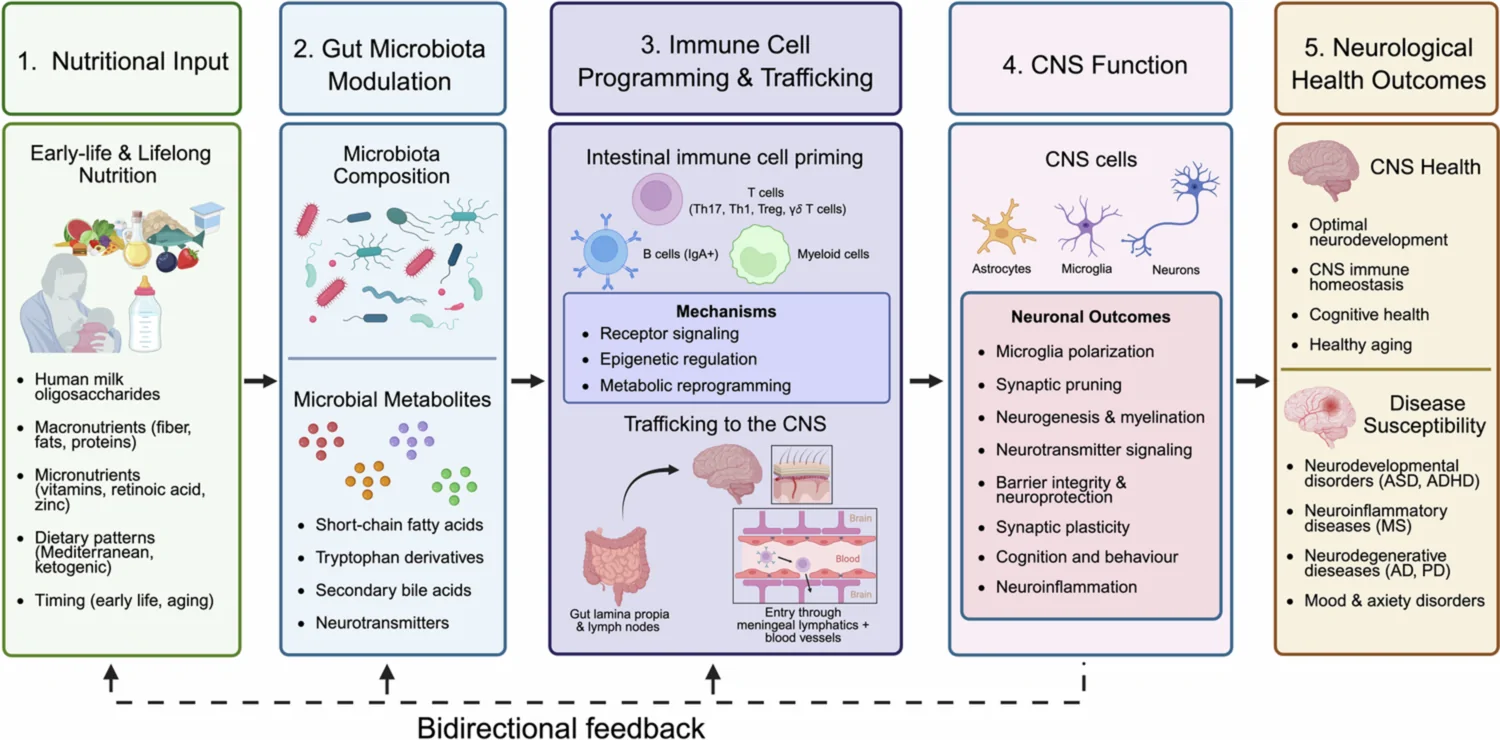
A hierarchical framework for nutritional modulation of health and disease across the lifespan.

**Figure 2.**
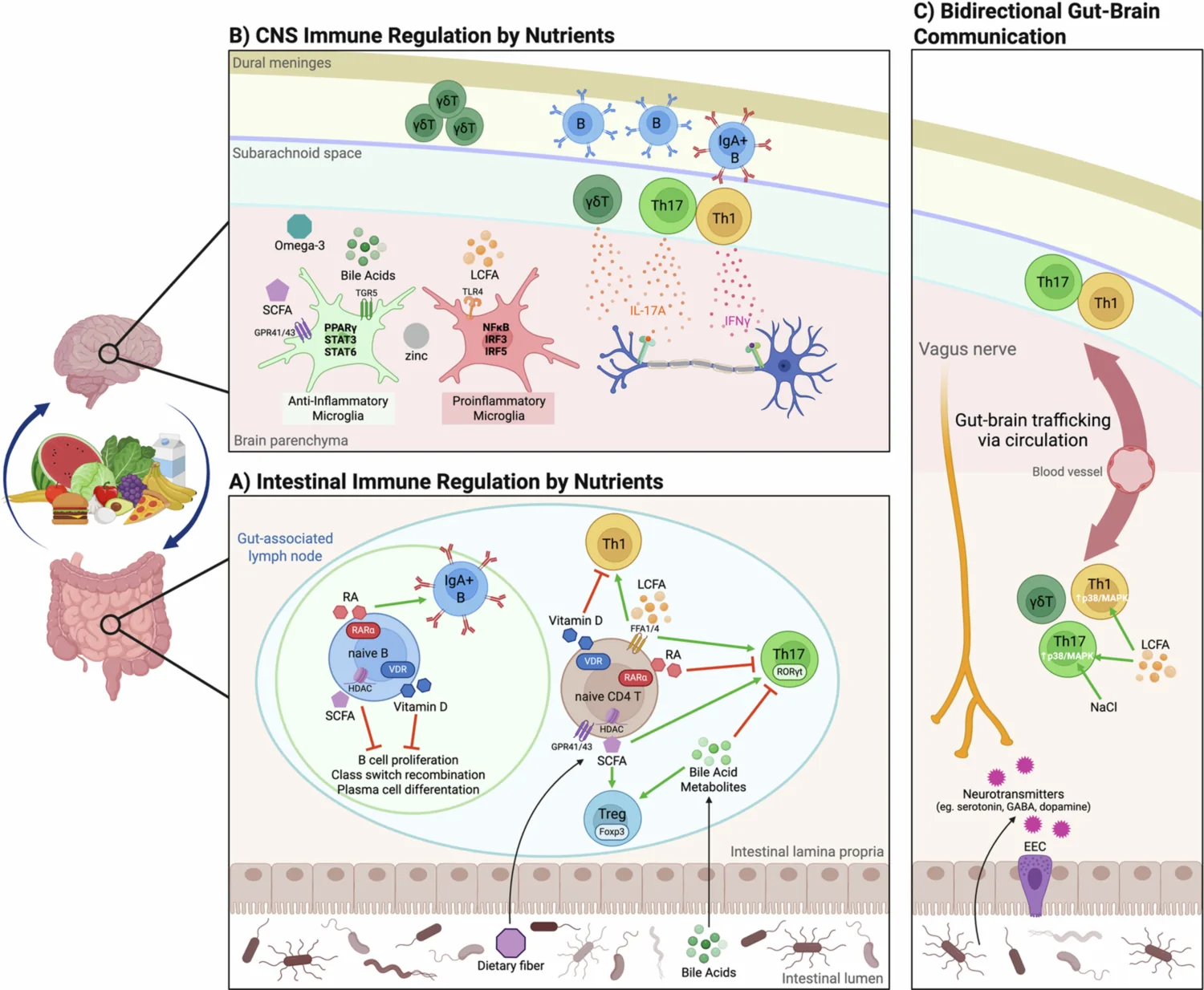
Nutrition regulates gut-brain immune crosstalk through local cell types and bidirectional signaling mechanisms.

The past decade of research has brought to light the pervasive role of the gut microbiota in shaping CNS immunity through multiple routes, including the entry of microbial metabolites into the CNS, priming of intestinal lymphocytes which traffic to the brain, and vagal afferent signaling. Importantly, diet sits upstream of these pathways and is well-positioned as a modifiable strategy for treating emerging neurological disorders. Recent reviews of the gut-brain-immune axis discuss nutrition as one of many environmental inputs,^1–3^ while parallel work examines how nutrient availability shapes microglial polarization without engaging the contributions of the gut microbiota.^4^ Thus, there is a need to synthesize knowledge focused on the role of host nutrition in shaping multiple facets of gut-brain-immune crosstalk. Here we propose a hierarchical framework in which nutritional inputs propagate through successive levels of microbial metabolism, immune cell programming, and neuronal function, to shape host physiology and disease outcomes (Figure 1). By applying this framework across the lifespan and in settings of health and disease, this review emphasizes how nutrition modulates gut-brain-immune interactions to shape CNS immunity and neurodevelopment (Figure 2 and Table 1). In highlighting these processes, we aim to elucidate the potential of nutritional interventions to optimize neuroimmune health and prevent disorders across the lifespan.

## 2. Regulation of CNS neuronal responses by immune cells

### 2.1. Microglia

Baseline immunity in the CNS is mediated by microglia, resident macrophages situated near neuronal synapses which promote synaptic pruning and remodeling,^5^ ^,^ ^6^ synaptic plasticity,^7^ ^,^ ^8^ and removal of apoptotic neurons during development.^9^ Microglia arise from embryonic myeloid progenitors and are among the first cells to populate the developing CNS, where they support early neurogenesis by secreting growth factors and guiding synapse formation.^10^ Phagocytosis of myelin debris and apoptotic cells by microglia, mediated by complement and fractalkine receptors (CX3CR1),^11^ ^,^ ^12^ is also crucial for maintaining brain homeostasis and removing neurotoxic factors; defects in this process are associated with neurodegenerative and neuroinflammatory disorders.^13^ In mice, diphtheria-mediated microglia depletion causes reductions in motor-learning-dependent glutamatergic excitatory synapses and impaired performance in learning tasks,^14^ further highlighting the central role of microglia in supporting homeostatic brain function and behavior.

In the setting of CNS disease, microglia conversely exert proinflammatory functions that drive neuroinflammation and neurodegeneration. Disease-associated microglia (DAMs) are transcriptionally distinct from their homeostatic counterparts, upregulating genes involved in interferon response, stress response, and lipid metabolism.^15^ As CNS disorders are more common later in life, DAM accumulation is in part driven by age-dependent changes in microglia metabolism, namely a shift from oxidative phosphorylation to aerobic glycolysis via a mTOR-HIF-1a-dependent pathway.^16^ This results in microglial lactate build-up, which promotes neuronal damage through acidification of the brain microenvironment and stimulates IL-6, TNF α , and IL-1 β production from microglia.^17^ ^,^ ^18^ Indeed, glycolysis is central to the proinflammatory functions of DAMs; glycolysis inhibitors (2-DG) have been effective in suppressing microglia-mediated proinflammatory cytokine production and neuroinflammation in mice.^19^ In mouse models of Alzheimer’s disease (AD) and Parkinson’s disease (PD), DAMs worsen disease progression by excessive phagocytosis-mediated synaptic pruning leading to neurodegeneration. In contrast, DAMs appear to be more neuroprotective in humans, as loss-of-function mutations in DAM-associated genes is correlated with heightened neurodegeneration,^20^ indicating that further mechanistic studies are needed to understand the discrepancies in microglia function between mice and humans.

Microglia respond to injury or nutritional changes in the CNS to modulate neuroinflammation. Omega-3 fatty acids eicosapentaenoic (EPA) and docosahexaenoic (DHA), both rich in seafood, modulate microglial polarization via PPARγ and NF-κB pathways, promoting anti-inflammatory effects;^21^ while saturated fatty acids induce pro-inflammatory microglial activation, contributing to neuroinflammation.^22^ In the absence of microbiota in germ-free (GF) mice, epigenetic changes in histone acetylation and methylation at gene promoter regions control proliferation, activation, and metabolic functions of microglia; microbiota-derived acetate restores mitochondrial metabolic insufficiency in GF microglia. However, a study using 5xFAD mice, a widely used transgenic mouse model for studying AD, showed that chronic oral administration of acetate increases Aβ deposition levels, effectively increasing hippocampal plaques that contribute to the development of AD.^23^

### 2.2. Adaptive immune cells

In addition to microglia, adaptive immune cells also play an essential role in maintaining CNS homeostasis. While largely absent from the homeostatic brain parenchyma of mice and humans, meningeal-resident and peripheral adaptive lymphocytes actively patrol the CNS through meningeal blood vessels and host diverse immune responses within the meninges. *Rag1* ^*-/-*^ mice, which lack mature T and B cells, exhibit deficits in hippocampal neurogenesis, reduced brain-derived neurotrophic factor (BDNF) production, impaired learning and memory, and increased anxiety-like behavior,^24^ ^,^ ^25^ further highlighting the indispensable role of adaptive lymphocytes in regulating neuronal responses and behavior.

In mammals, the transient migration of CD4 T cells into the brain around birth is necessary for microglia maturation and synaptic pruning;^26^ adoptive transfer of CD4 T cells is sufficient to rescue cognitive and behavioral defects observed in *Rag1* ^*-/-*^ mice.^24^ ^,^ ^25^ While CD4 T cells can directly interact with neurons, their neuromodulatory effects are predominantly mediated by cytokines which signal through cognate receptors expressed on neurons and glia. IL-4 was among the first cytokines shown to regulate cognition in mice by inducing astrocytic production of BDNF to promote learning and memory.^27^ More recently, IFN γ has been described to regulate social behavior in mice through signaling to interneurons and modulating GABA production.^28^ *Ifng* ^-/-^ mice exhibit social deficits that can be rescued by repopulation with wildtype, but not *Ifng* ^*-/-*^, T cells.^28^ Additionally, IL-17A has been shown to regulate offspring neurodevelopment following maternal immune activation in mice, where CD4 T cell-derived IL-17A promotes cortical malformations and autism-like behaviors in offspring.^29^

The murine dural meninges is mostly composed of B cells derived from and locally replenished by the skull bone marrow, which may facilitate cellular and humoral tolerance to CNS antigens.^30^ ^,^ ^31^ Notably in mice, the steady state meninges also harbors a small population of IgA + plasma cells which are believed to originate from the intestines.^32^ In mouse models of neuroinflammation or cancer, meningeal B cells form follicle-like structures that locally produce antibodies against CNS antigens, with multifaceted effects on disease progression.^33^ The meninges also harbors resident γδ T cells which regulate synaptic plasticity, short-term memory, and anxiety-like behavior in mice through production of IL-17A.^34^ ^,^ ^35^ On the regulatory side, meningeal Tregs are specialized in controlling IFN γ responses within the CNS; loss of meningeal Tregs leads to defects in hippocampal neurogenesis and memory in mice.^36^

Open questions pertaining to the regulation of neuronal responses by the immune system include the antigen specificity of meningeal T and B cells, the signals that mediate immune cell trafficking to the CNS under homeostatic conditions, and the regulation of their activation once within the CNS environment.

### 2.3. Immune regulation via the gut-brain axis

The gastrointestinal tract and gut microbiota have emerged as key regulators of CNS neuronal responses through a bidirectional communication system involving neuronal, endocrine, and immune mediators. Immune cells are important facilitators of gut-brain communication, acting both systemically and locally within the gut and brain. The gut provides an antigen-rich environment to prime peripheral immune cells that can traffic to the brain, which increases dramatically during neuroinflammation or following brain injury. Indeed, intestinal Th17 cells and IgA+ plasma cells traffic to the spinal cord during Experimental Autoimmune Encephalomyelitis (EAE) in mice,^37^ ^,^ ^38^ while intestinal γδ T cells infiltrate the leptomeninges in models of ischemic stroke.^39^ It is further speculated that gut antigens may function as molecular mimics of CNS antigens, whose accessibility to prime the peripheral immune system remains debated, to prime CNS-specific immune responses. One such study found that *Lactobacillus reuteri* contains peptides which mimic those found in myelin oligodendrocyte glycoprotein (MOG) and could stimulate MOG-reactive T cells to worsen EAE.^40^ Gut microbiota composition is further known to polarize intestinal immune responses in mice and subsequently systemic or CNS immunity. For instance, colonization of GF mice with segmented filamentous bacteria (SFB), which is known to induce Th17 cells in the small intestine,^41^ also increases Th17 cells in the CNS and exacerbates EAE severity.^42^

Increased proinflammatory maternal immune responses, which can be elicited by infection during pregnancy, has also been linked to impaired fetal brain development and offspring neurodevelopmental disorders in mice.^43^ Seminal research in mice revealed maternal Th17 cells to be involved in this process, wherein IL-17A produced by Th17 cells enters the fetal brain to induce abnormal cortical architecture that increases autism-like behavior in adulthood.^29^ The maternal gut microbiota is implicated in maternal immune activation, as the absence of SFB in pregnant dams led to reductions in maternal Th17 cells and could rescue behavioral phenotypes in offspring.^44^

## 3. Microbial regulation of CNS neuroimmunity

### 3.1. Gut bacteria

Microbial stimuli are required for maturation of both the immune and nervous systems, as highlighted by observations that GF mice have underdeveloped secondary lymphoid structures,^45^ altered neuronal activity in the brain, and changes in social behavior.^46^ Several studies have further demonstrated that the gut microbiota is required for neurodevelopmental processes such as blood-brain barrier (BBB) formation and integrity,^47^ microglia maturation,^48^ ^,^ ^49^ and CNS myelination in mice.^50^

Gut bacteria affect brain health largely through the production of soluble factors, such as microbial metabolites and neurotransmitters, which can act locally in the intestines via the vagus nerve or enter the bloodstream and traverse the BBB to signal directly to neurons and glia. In addition to these soluble mediators, discussed below, emerging research highlights the importance of overall gut bacteria composition and ecosystem stability to maintaining host physiology. Individual bacterial species shape CNS immunity and brain health through induction of proinflammatory or regulatory immune responses (SFB: Th17, *B. fragilis*: Tregs),^41^ ^,^ ^51^ maintenance of intestinal barriers to limit systemic spread of inflammation, and direct stimulation of the vagus nerve. Bacteria interaction networks and ecosystem stability also contribute to the overall intestinal milieu and immune priming across the gut-brain axis; perturbations from diet, antibiotics, or inflammation induce gut dysbiosis which often results in pathobiont overgrowth at the expense of beneficial species. Next-generation sequencing techniques have permitted increased study of microbial network dynamics and community structure,^52^ whose role in regulating host physiology and gut-brain immunity is now beginning to be appreciated. In addition to composition of the gut microbiota, its flux and the community's capacity to maintain homeostasis under perturbation determines neuroimmune tone and, plausibly, susceptibility to neurological disease.

### 3.2. Microbial metabolites

#### 3.2.1. Microbial products of dietary nutrients

Metabolism of dietary nutrients by the gut microbiota generates essential metabolites with diverse immune and neuro-modulatory functions. Notably, short-chain fatty acids (SCFAs), namely acetate, propionate, and butyrate, generated from microbial fermentation of dietary fiber have well-described functions in regulating T cell and innate lymphoid cell responses in mice.^53^ SCFAs can also traverse the BBB and bind to free fatty acid receptors (FFAR) on microglia to directly modulate microglial processes.^48^ Indeed, oral administration of SCFAs to GF mice rescues microglia growth defects, promotes microglia transcriptional maturation, and recovers BBB integrity.^47^ ^,^ ^48^ Mechanistically, SCFAs are potent inhibitors of histone deacetylase and can enhance transcription of genes associated with synaptic plasticity in neurons and microglia, or anti-inflammatory genes in naïve T cells; these effects have been extensively reviewed elsewhere.^54^

Tryptophan, an essential amino acid derived from dietary protein is another important substrate for microbial metabolism, generating aryl hydrocarbon receptor (AhR) agonizts, such as indole derivatives, which can also traverse the BBB to directly regulate microglia-astrocyte crosstalk.^55^ AhR agonizts induce TGF and VEGF- β production from microglia, which in turn regulates the transcriptional program of astrocytes in mouse models of neuroinflammation.^55^ Tryptophan can also be metabolized to kynurenine, which suppresses inflammation by signaling through GPR35 expressed on immune cells and has been shown to suppress EAE symptoms in mice.^56^ ^,^ ^57^ Aging-driven gut dysbiosis and systemic inflammation increases activity of the kynurenine pathway mediated by host indoleamine 2,3-dioxygenase (IDO) in mice; this reduces tryptophan availability and increases neurotoxic metabolites like quinolinic acid (derived from tryptophan via IDO-kynurenine pathway).^58^ ^,^ ^59^ This also significantly limits the availability of gut bacteria-derived AhR agonizts and their signaling to CNS microglia and T cells.

#### 3.2.2. Microbial products of host molecules

Primary bile acids (e.g., cholic acid, chenodeoxycholic acid) are synthesized from cholesterols in the liver and are then converted by gut bacteria into secondary bile acids, which signal through farnesoid X receptor (FXR) and G-protein-coupled bile acid receptor (TGR5) expressed on immune and glial cells.^60^ Secondary bile acids, such as lithocholic acid derivatives (e.g., 3-oxo-LCA), promote Treg differentiation and inhibit Th17 cell polarization in mice by directly binding to retinoid-related orphan receptor gamma t (RORγt), a transcription factor critical for Th17 cells.^61^ ^,^ ^62^ Bile acids can cross the BBB to activate microglia and astrocytes, triggering inflammatory cascades via pathways like NF-κB, leading to release of IL-1 β and TNF α in the CNS. Thus, gut microbiota-mediated increases in secondary bile acids, such as DCA or 3-oxo-LCA, may potentially reduce pro-inflammatory T cell (Th17, Th1) infiltration into the brain and dampen CNS neuroinflammation. Indeed, elevated levels of bile acids such as taurocholic acid in humans are linked to increased neuroinflammation in hepatic encephalopathy and AD.^63^ Bile acid metabolism is also altered in Multiple Sclerosis (MS), and supplementation of tauroursodeoxycholic acid (TUDCA) to mice prevented polarization of astrocytes and microglia to neurotoxic phenotypes and ameliorated neuropathology in EAE.^64^

### 3.3. Regulation of neurotransmitters by the gut microbiota

The gut is a rich source of neurotransmitters produced by enterochromaffin cells (EECs) and bacteria, generating up to 90% of the body’s total serotonin and 50% of dopamine. Gut bacteria are necessary for neurotransmitter production, as GF and antibiotic-treated mice exhibit changes in fecal and serum neurotransmitter levels, as well as altered neurotransmitter receptor expression in the brain.^65^ ^,^ ^66^ Gut bacteria contribute to neurotransmitter generation directly, such as through *Lactobacillus* production of GABA, dopamine, catecholamines, and histamine, or indirectly, such as through *Clostridia* indirectly promoting serotonin biosynthesis by increasing gene expression of the rate-limiting enzyme tryptophan hydroxylase-1 in murine colonic EECs.^67^ Gut-derived neurotransmitters influence CNS immunity through the vagus nerve or intestinal immune cells. For instance, serotonin enriched in the mouse neonatal intestine promotes Treg induction and is essential for proper development of immune tolerance in early life.^68^ Maintenance of intestinal immune homeostasis protects against neuroinflammation by preserving barrier integrity and limiting trafficking of immune cells and inflammatory mediators. It was also recently shown that activation of dopamine receptors on intestinal epithelial cells exacerbated EAE severity in mice by regulating abundance of gut bacteria that affect microglia activation.^69^

Diet regulates neurotransmitter synthesis not only through shaping gut microbiota composition, but also by supplying essential amino acids that the host cannot endogenously produce, such as tyrosine, tryptophan, and glutamine, which act as precursors for the synthesis of dopamine, serotonin, and GABA in both the gut and brain. Amino acids are derived from dietary protein and low-protein diets have been associated with behavioral abnormalities and low neurotransmitter production in mice,^70^ while higher protein intake is associated with reduced risk of cognitive decline in healthy adults.^71^ Dietary nutrients can further modulate neurotransmitter production by EECs in mice, as luminal sugars have been found to activate small intestine EECs to synapse with vagal afferents and produce glutamate to signal to the brain.^72^

### 3.4. The vagus nerve

The vagus nerve, which extends from the brainstem to gastrointestinal tract, is a primary conduit for bidirectional communication between the gut and brain. The enteric nervous system sends sensory signals (such as nutrient status) via vagal afferents to the CNS, modifying neurotransmitter production and signaling within the CNS, which then responds with vagal efferent signals to modulate gut motility and intestinal immune responses.^73^ Gut bacteria can directly modulate neuronal function through the vagus nerve, demonstrated by a seminal mouse study which found that continuous oral treatment with *Lactobacillus rhamnosus* (JB-1) could alter expression of GABA receptors in the brain and reduce depression-like behavior, effects which were abolished in vagotomized mice.^74^ Vagus nerve activation in the intestines, by electrical gradients, nutrients, hormones, or cytokines, leads to release of neuroactive factors that alter neuronal firing and microglia activation in the CNS.^75^ For instance, it was demonstrated in mice that luminal glucose sensed by EECs stimulates their release of glutamate and activation of vagal afferent fibers that communicate with brain neurons involved in appetite regulation.^76^ ^,^ ^77^

Beyond the intestine, the vagus nerve is critical for whole body-brain interoception, with vagal sensory neurons integrating diverse signals across the periphery and transmitting them to the CNS. In mice, these sensory signals are encoded across three independent dimensions that combine to specify parallel signaling pathways, allowing precise coding of the body’s complex internal state.^78^ These vagal pathways are now appreciated to have an essential role in sensing and regulating peripheral inflammation. Notably, in mice, action potentials in the vagus nerve stimulate splenic T cells to produce the neurotransmitter acetylcholine, which dampens macrophage proinflammatory responses via the α7 nicotinic acetylcholine receptor.^79^ Similarly, in rats, peripheral vagus nerve stimulation during lethal endotoxaemia induces the production of acetylcholine which signals through nicotinic acetylcholine receptor α7 expressed on intestinal macrophages to limit their production of TNFα, IL-1 β , and IL-6 and protect against septic shock.^80^ Vagotomy in rats is sufficient to increase serum levels of TNF, further suggesting that efferent vagus activity directly regulates TNF production and exerts tonic suppression of peripheral inflammatory responses. The efferent vagus nerve also regulates localized peripheral inflammation, as electrical stimulation of the distal vagus nerve inhibits localized inflammation in a rodent model of carrageenan-induced paw edema.^81^ Dietary nutrients appear to be important regulators of this cholinergic anti-inflammatory pathway, as acetylcholine release from vagal efferents can be stimulated by high-fat diet-induced production of cholecystokinin (CCK) in rats.^82^

Permanently implanted vagus nerve stimulators are clinically approved devices for the prevention of seizures in epileptic patients through activation of vagal afferents associated with limbic and cortical function.^83^ The cholinergic anti-inflammatory pathway activated by vagal stimulation further presents a novel therapeutic avenue to treat neurological disorders through suppression of peripheral inflammation, which is frequently associated with clinical pathology.^84^ Moreover, the ability of the gut microbiota and dietary nutrients to stimulate vagal afferents provides impetus for developing non-invasive therapeutic methods of vagus nerve stimulation, such as through selective probiotics or dietary intervention.

## 4. Nutrition & neuroimmmunity across the lifespan

### 4.1. Nutrients implicated in neuroimmunity

Among dietary micronutrients, vitamins are essential for immune cell development and function. The primary metabolite of vitamin A, retinoic acid, signals through retinoic acid receptors expressed on immune cells and plays an important role in regulation of CD4 T cell responses. Notably, retinoic acid inhibits IL-6-driven Th17 cell differentiation and promotes the generation of Foxp3+ Tregs in mice,^85^ while dendritic cells in gut-associated lymphoid tissue also enhance peripheral Treg generation in a retinoic acid-dependent manner.^86^ This immunoregulatory capacity of retinoic acid may further extend to the CNS, as intestinal Tregs have been reported to traffic to the brain in mouse models of ischemic stroke.^35^ ^,^ ^77^ Another essential vitamin, Vitamin D, has been shown to be a potent inhibitor of T and B cell proliferation, plasma cell differentiation, and IgG secretion in mice.^87^ In the context of neuroimmunity, vitamin D has been shown to inhibit EAE progression in mice by blocking generation of Th1 cells which invade the CNS and drive neuroinflammation through IFN γ production.^88^ Similarly, antioxidant vitamins, such as vitamins E, C, and B6, have broad anti-inflammatory effects through inhibition of reactive oxygen species, which may be clinically relevant for treatment of autoinflammatory CNS and gut disorders.

Zinc is another micronutrient important for regulating neuroimmunity, as nearly 16% of the human body’s total zinc can be found within the CNS. Zinc has been described to have both neurotoxic and neuroprotective effects within the CNS of mice, both of which occur through modulation of microglia. While some studies report increased production of inflammatory cytokines from rat immortalized microglia cells treated with zinc,^89^ others find the opposite effect via downregulation of NFkB.^90^ *In vivo*, treating mice with a zinc chelator reduces EAE severity while zinc supplementation reduces neuroinflammation and improves motor function in a mouse model of spinal cord injury.^91^ ^,^ ^92^ Thus, the role of zinc in regulating microglia function is likely dynamic and dependent on existing immune signals or inflammation within the CNS.

### 4.2. Early life nutrition

Sensitive time windows during host development are vulnerable periods that, if disturbed, can lead to irreversible alterations in proper growth and function of the body. During these periods, the composition of the gut microbiota exhibits the greatest instability and variability, often leading to disruptions in immune cell function and neuronal signaling. Thus, the quantity and quality of dietary nutrients received during these sensitive time windows is a critical determinant of host microbiota composition, CNS development and homeostasis, and immune system priming across the lifespan.

The human brain is highly metabolically active during postnatal development, with its energy expenditure accounting for half the daily total resting energy metabolism.^93^ Parallel to early neurodevelopment, the formation of the gut-brain axis also begins in early life, following initial colonization of the intestines by gut bacteria at birth, thus making early life nutrition a critical determinant of future brain health. Breast milk is the primary source of dietary micro- and macronutrients during early life, but its composition is highly variable and influenced by the mother’s diet, mental health, and overall lifestyle. Notably, breast milk is rich in bioactive components such as human milk oligosaccharides (HMOs). Fucosylated HMOs, especially 2’-FL, predominate in human breast milk and have been demonstrated to enhance hippocampal long-term potentiation and learning and memory in rats.^94^ ^,^ ^95^ The neuroprotective effects of HMOs may depend on microglia, wherein 2’-FL has been shown to reduce accumulation of reactive oxygen species and promote STAT6-dependent M2 microglia polarization in a mouse model of ischemic stroke.^96^ Mechanistically, a recent study in which radiolabeled 2’-FL was orally administered to mice revealed that 2’-FL itself does not cross the BBB or become incorporated into brain structures, but may be used to generate neuroactive metabolites through bacterial fermentation.^97^ Another study similarly found that oral administration of HMOs significantly attenuated necrotizing enterocolitis (NEC)-induced brain injury in newborn mice and this was associated with the accumulation of 2’-FL metabolites and BDNF in the murine infant brain.^98^ In humans, HMOs are also known to broadly promote microbial diversity and improve barrier function in the infant gut, and may thus indirectly influence neurodevelopment through microbial mechanisms of gut-brain regulation.^99^ ^,^ ^100^

The introduction of solid food is a critical event for maturation of the mammalian infant immune system and gut microbiota. This “weaning reaction” in mice is necessary for generation of immune tolerance against food antigens through the induction of peripheral RORγt+ Tregs.^101^ In mice, RORγt+ Tregs are known to inhibit intestinal inflammation as well as Th17 cell-mediated CNS autoinflammation.^102^ ^,^ ^103^ Recently, dietary tryptophan deficiency was found to induce expansion of intestinal RORγt+ Tregs in mice,^104^ revealing another pathway through which nutrition can potentially modulate gut-brain-immune crosstalk. Early weaning in mice consistently increases anxiety-like behaviors,^105^ ^,^ ^106^ aggression,^107^ and delayed conditioned-fear extinction in adulthood,^108^ partially driven by decreased neural connectivity and BDNF production in the brain,^105^ although this effect is likely caused by psychological stress induced by maternal separation rather than changes in infant nutrition.

### 4.3. Mid-life nutrition & sex differences

The transition to puberty is marked by an initiation of sexually dimorphic processes, including changes in the gut microbiota. Castration of male mice during the pubertal window eliminates sex differences in gut microbiota composition in adulthood, demonstrating an important role for pubertal testosterone in organizing sexually dimorphic microbial communities,^109^ however, the exact mechanism for androgen-mediated regulation of gut bacteria is not yet known. Notably, transfer of male fecal matter is sufficient to increase testosterone production in female mice and confer disease protection in a murine model of type 1 diabetes, which is female-biased.^109^ It is not yet known whether sex-specific microbiome transfers may impact sex differences in neurological or autoinflammatory diseases through the modulation of gonadal hormones. The brain also undergoes sex-specific development throughout puberty,^110^ which may be further influenced by gut bacteria-induced changes in gonadal hormones and neurotransmitters. Thus, nutrition during puberty and mid-life may contribute to sex-specific brain development and function through the gut microbiota.

In humans, sex-specific alterations of gut microbiota diversity and composition, especially in adolescence and adulthood, have been reported and extensively reviewed.^111^ Certain bacterial taxa are differentially abundant in males and females during adulthood, such as females having more *Ruminococcaceae*, *Faecalibacterium*, and *Alistipes*, while males have more *Prevotella*.^112^ These differences in gut microbiota composition may differentially affect nutrient availability and absorption, gut barrier functions, immune cell priming, and neurological function in males and females throughout adolescence and adulthood. Further, sex-specific changes in gut microbiota composition in response to environmental factors, such as infection and stress, may contribute another layer of regulation in terms of sex differences in neurological disease risk.

### 4.4. Nutrition & aging

Aging profoundly impacts the function of immune cells in the brain, including microglia and adaptive lymphocytes, contributing to autoimmunity or chronic inflammation (“inflammaging”), and increased susceptibility to CNS autoimmunity or neurodegenerative diseases, respectively. Relevant to nutrition and immunity, the aged human gut microbiota also exhibits reduced microbial diversity, greater instability, and loss of beneficial bacterial species with a simultaneous increase in gut pathobionts.^113^ Functionally, these microbiota changes result in reduced synthesis of SCFAs and essential amino acids,^113^ necessitating their supplementation in the diet during old age. The aged gut microbiota in mice has been shown to induce markers of cellular senescence in gut germinal center B cells and reduce IgA responses,^114^ suggesting that gut bacteria also contribute to modulation of immune function with age.

#### 4.4.1. Microglia

The CNS adopts a more inflammatory profile with age, a process associated with heightened microglia sensitization. Microglia from aged humans and mice exhibit heightened expression of MHC II, indicative of antigen-experienced priming, and other inflammatory markers such as CD86, toll-like receptors, IL-1β, and indoleamine 2,3-dioxygenase.^115^ ^,^ ^116^ In aged mice, peripheral LPS administration promotes sustained microglia activation and downregulation of IL-4 receptor expression, which is associated with reduced responsiveness to the M2-polarizing effects of IL-4.^117^ Thus, aged microglia appear to specifically retain an inflammatory profile and become less sensitive to anti-inflammatory stimuli. Aged microglia also have reduced phagocytic capacity, leading to the accumulation of excess soluble factors in the brain that further drives neuroinflammation and CNS dysfunction with age.^118^

Shifts in microglia metabolism with age likely contribute to their altered functionality, where aged microglia from mice preferentially rely on glycolysis for energy production while having reduced mitochondrial efficiency and impaired autophagy.^119^ This reduces microglia ATP production while promoting the accumulation of reactive oxygen species which may potentiate the inflammatory phenotype of aged microglia. To this end, dietary nutrients may regulate microglia function during aging by affecting cell metabolism. In both humans and mice, consumption of a high fat diet is linked to greater cognitive decline and enhanced microglial activation with age.^120^ Saturated fatty acids directly induce microglia production of IL-1β, which signals to hippocampal neurons and impairs learning and memory in rats.^121^ ^,^ ^122^ Conversely, antioxidant nutrients such as vitamin E, flavonoids, and omega-3 PUFAs, have been found to improve cognitive function in elderly individuals and may prevent inflammatory microglia polarization by scavenging reactive oxygen species, however this mechanism has not been definitively tested.^120^

#### 4.4.2. T cells

T cell infiltration into the CNS increases with age and is driven by age-related increases in BBB permeability and chemokine induction in the proinflammatory CNS environment.^123^ Within the aged CNS, T cells may functionally contribute to age-related cognitive decline through various mechanisms. Memory Th2 cells have been found to accumulate in the choroid plexus of aged mice and produce IL-4 leading to induction of CCL11 expression from brain epithelial cells and continued recruitment of immune cells into the CNS.^124^ Interestingly, restoration of the Th1/Th2 balance through induction of peripheral T cell proliferation partially rescued plasticity-related neuronal gene expression in aged mice,^124^ suggesting that age-dependent T cell senescence contributes to cognitive decline by shifting the balance of CD4 T cell responses. More recently, CD8 T cells have also been found to accumulate in the brain parenchyma of aged mice. The production of granzyme B and IFN γ by CD8 T cells directly triggers neuronal axon degeneration and alters oligodendrocyte function leading to reductions in myelination.^125^ ^,^ ^126^

The metabolic state of CNS T cells is profoundly affected by aging as well, characterized by impaired oxidative phosphorylation (OXPHOS) and increased dependence on glycolysis; this leads to T cell senescence and reduced proliferation and effector function, which weakens CNS immune surveillance. Impaired T cell survival and function with age is directly associated with loss of telomerase activity, accumulation of DNA damage, and shifts in cellular metabolism towards reduced production of lactate and ATP.^127^ These metabolic shifts further favor pro-inflammatory T cell subsets (e.g., Th17 or Th1 cells), contributing to chronic inflammation in neurodegenerative diseases.^128^ ^,^ ^129^

#### 4.4.3. B cells

In mice and humans, aging reduces naïve B cell generation in the bone marrow and is associated with systemic reductions in germinal centers and antibody responses. These defects are driven in part by age-related alterations in B cell metabolism, as aged B cells have reduced glycolytic capacity and OXPHOS and cannot meet the energetic demands for germinal center initiation and antibody production, both of which depend heavily on glycolysis.^130^ ^,^ ^131^ In the gut, loss of IgA antibodies with age contributes to gut microbiota dysbiosis in mice,^114^ which can lead to increased inflammatory signaling across the gut-brain axis driving neuroinflammation. Age-dependent changes in B cell metabolism also promote the generation of age-associated B cells (ABCs) in both mice and humans, which are characterized as having a myeloid-like phenotype.^132^ ^,^ ^133^ ABCs accumulate in the dural meninges of aged mice and contribute to CNS autoimmunity and neurodegeneration through production of autoantibodies against CNS antigens. While the mechanisms promoting ABC recruitment to the aged CNS remain unclear, the age-dependent accumulation of inflammatory mediators in the CNS is an important pathway that can be targeted by optimizing host nutrition with age.

#### 4.4.4. Optimizing nutrition with age

Nutrition may help combat age-associated declines in CNS immune function. First, adequate macronutrient and micronutrient quantities are necessary to support the changing energetic demands of immune cells, as malnutrition in elderly individuals intensifies immune decline and susceptibility to infection.^134^ In addition, antioxidants such as vitamin E are important to combat increased oxidative stress and accumulation of free radical damage that occurs with normal aging and can contribute to inflammation in the CNS.^135^ ^,^ ^136^ Studies of centenarians, individuals over the age of 100 who are resistant to the development of age-associated diseases, may reveal mechanisms of age-associated immunosenescence and highlight nutritional strategies for preserving immune function with age. Centenarians are generally resistant to the immune changes described below and exhibit a reduced systemic Th17:Treg ratio that may broadly protect against neuroinflammation and inflammaging.^137^ Similarly, aged adults who retain strong cognitive function (‘SuperAgers’) were shown in a recent study to exhibit greater hippocampal neurogenesis;^138^ this study further found that neuronal epigenetic differences represent more distinctive signatures of age-associated cognitive decline than gene expression.^138^ These findings highlight the importance of environmental contributions, such as diet and nutrition, to healthy CNS aging, in part by epigenetic imprinting. In line with this, studies of centenarians reveal associations between plant-forward diets and healthy immune aging.^137^ Interestingly, the centenarian gut microbiome resembles that of young individuals observed under caloric restriction.^137^

## 5. Nutrition & disease

Various neurological and neuropsychiatric disorders in humans are associated with changes in gut microbiota composition, implicating the use of diet and nutrition-based modification of the gut microbiome as a viable therapeutic strategy. While preclinical studies in animal models of neurological disease have begun to elucidate the mechanisms by which host nutrition shapes gut microbiota and immune responses to impact disease outcome, the application of this knowledge towards large-scale clinical trials of nutritional interventions in human patients remains limited.

### 5.1. Neuroinflammatory diseases

In MS, infiltrating leukocytes and aberrant microglia activation promote demyelination of neuronal axons. CD4 T cell infiltration into the CNS and their production of inflammatory cytokines is a key pathogenic mechanism of MS which can be modulated by dietary nutrients. For instance, long-chain fatty acids (LCFAs) commonly found in processed foods promote differentiation of naïve T cells into proinflammatory Th1 and Th17 cells in a MAPK/p38-dependent manner.^139^ In mice, consumption of a LCFA-rich diet leads to more severe EAE as a result of increased infiltration of Th1 and Th17 cells into the CNS.^139^ Conversely, consumption of SCFAs, which promote Treg generation, leads to less severe EAE in mice.^139^ Omega-3 PUFAs, such as DHA and EPA, also promote regulatory immune responses by signaling downstream nuclear receptors, metabolic cell reprogramming, and modulation of immune cell membranes. Accordingly, consumption of PUFAs by mice leads to less severe EAE with reduced production of inflammatory cytokines and enhanced remyelination.^140^ ^,^ ^141^ Despite these promising preclinical findings, clinical trials using PUFAs for treatment of MS have yielded inconsistent results.^142^ ^,^ ^143^ Several studies have further suggested neuroinflammatory effects of a high salt diet, which induces serum glucocorticoid kinase 1 expression and stabilizes IL-23 signaling to promote differentiation of pathogenic Th17 cells.^144^ Mice fed a high salt diet exhibit more severe EAE due to enhanced generation of pathogenic Th17 cells.^145^ In humans, clinical studies have shown that salt intake does not affect MS susceptibility in pediatric and adult populations,^146^ ^,^ ^147^ but one observational study found that high salt intake was associated with increased clinical severity in people with pre-existing MS.^148^ These discrepancies may reflect the ability of high sodium-induced pathogenic Th17 cells to worsen the progression, rather than initiation, of neuroinflammation.

### 5.2. Neurodegenerative diseases

Early mouse studies established a critical role for the gut microbiota in AD pathogenesis. Notably, mice genetically predisposed to beta-amyloid (Aβ) precursor protein mutations (APP mice) and lacking a conventional microbiota have dramatically reduced cerebral Aβ amyloid deposition, one of the key markers of AD;^149^ recolonization of GF APP mice with a conventional microbiota is sufficient to restore amyloid pathology. Similar studies focused on altering the gut microbiota using long-term broad-spectrum antibiotic treatment, which was found to significantly reduce cerebral Aβ plaque deposition in APP mice, while increasing levels of soluble Aβ peptides.^150^ Antibiotic treatment was accompanied by altered inflammatory signaling, reduced activation of microglial and astrocytic cells, and varied cell morphology in response to the absence of plaques.^150^ Together, these studies highlight the gut microbiota as a key modulator of AD pathology and call for future studies to investigate its modulation as a therapeutic strategy for disease treatment.

In terms of specific nutrients or gut metabolites implicated in AD pathogenesis, numerous studies have reported on the protective effects of SCFAs. Butyrate administration to AD mice has been shown to slow cognitive impairment, reduce Aβ plaques, and promote microglia maturation—reviewed here.^151^ Notably, one study counterintuitively demonstrated that SCFA supplementation in GF AD mice restored amyloid pathology,^152^ suggesting that the effects of SCFAs on microglia and AD pathology may be dependent on the overall gut microbiota ecosystem. Despite preclinical success in treating the clinical symptoms of AD in mice using sodium butyrate or SCFA-producing probiotic bacteria,^153^ clinical trials in human AD patients remain limited. Moreover, the use of dietary interventions to increase SCFA levels, such as high-fiber diets, may represent a promising non-invasive therapeutic strategy for future trials of AD patients.

### 5.3. Neurodevelopmental diseases

Attention-deficit hyperactivity disorder (ADHD) is a common psychiatric disorder in young adults and is associated with reductions in dopamine synthesis and signaling in the CNS. Emerging evidence suggests that gut microbial metabolism may contribute to ADHD through alterations in dopamine production. A human study linking the gut microbiome to neuronal function in ADHD found minor alterations in the composition of the microbiome, including an increase in *Bifidobacterium*, in adolescents and adults with ADHD compared to healthy controls.^154^ *Bifidobacterium* enrichment was associated with greater predicted abundance of the bacterial genes encoding cyclohexadienyl dehydratase, an enzyme involved in the synthesis of phenylalanine, a major precursor of dopamine.^154^ The increased predicted ability of the microbiome to produce phenylalanine was correlated with reduced ventral striatal activation during reward anticipation, a well-established characteristic of ADHD patients.^154^ Interestingly, a separate randomized-controlled trial found that administering a micronutrient blend to 17 male children with ADHD for 10 weeks resulted in a significant decrease in *Bifidobacterium*.^155^ Micronutrient-induced reduction of *Bifidobacterium* was linked with decreased symptomatic scoring,^155^ corroborating previous reports of a functional role for *Bifidobacterium* in ADHD through modulation of dopamine synthesis. While these trials are limited by cohort size, the findings suggest that alterations in gut microbiota composition and metabolism may influence how dopamine affects ventral striatal activation via the gut–brain axis. In addition to the gut microbiota, nutrients such as iron and zinc function as cofactors for dopamine synthesis; their dietary supplementation may potentially benefit ADHD patients.

### 5.4. Comorbid neuroinflammation and intestinal inflammation

Intestinal and CNS inflammation often co-occur in patients with inflammatory bowel disease (IBD) or CNS disorders. For instance, children with autism spectrum disorder (ASD) are 6-8 times more likely to suffer from chronic gut or digestive conditions.^156^ Dysregulation of gut microbiota-derived metabolites is implicated in children with ASD, as levels of 5-aminovaleric acid (5AV) and taurine, weak GABA receptor agonizts, were found to be lower in children exhibiting autistic-like behavior.^157^ Furthermore, IBD patients are at higher risk of central and peripheral nervous system demyelination and cognitive impairments;^158^ this is in part due to dysregulation of the gut-brain axis by increased systemic inflammation and gut dysbiosis.^158^ Nutrient deficiencies (in vitamin D, B vitamins), which are common in IBD and MS, impair neurotransmitter synthesis and BBB integrity, promoting CNS inflammation.^159^ ^,^ ^160^ Disruption in the Th17/Treg balance is a pathogenic mechanism implicated in both diseases and may be the mechanistic link for vitamin D, which has been previously shown to promote Treg generation while suppressing Th17 differentiation.^161^ In addition, poor nutrition or gut dysbiosis can worsen “leaky gut” in IBD and MS, allowing inflammatory mediators to enter systemic circulation and increase CNS inflammation. Thus, many of the aforementioned nutrients implicated in CNS regulation are also important for maintaining intestinal homeostasis. Omega-3 PUFAs, for instance, help resolve gut inflammation in mice by improving gut barrier integrity,^162^ modulating gut microbiota composition,^163^ and inducing Foxp3+ Treg generation.^164^

## 6. Nutritional interventions to modulate gut-brain immune crosstalk

### 6.1. Dietary modifications

#### 6.1.1. The mediterranean diet

The Mediterranean diet is widely regarded as an optimal dietary pattern for improving immune and nervous system health. Its emphasis on fruits and vegetables provides plentiful vitamins, antioxidants, and minerals needed for optimal function of adaptive lymphocytes and microglia within the CNS. This is especially important in old age as nutrient requirements change with the shifting metabolic demands of immune cells. Indeed, several clinical trials have found that consumption of a Mediterranean diet by elderly individuals reduces risk for cognitive decline.^165–167^ In addition, caloric restriction, wherein energy intake is reduced by 30–40%, is also emerging as a means to combat age-related cognitive decline by promoting the clearance of waste in the CNS via autophagy. While clinical trials in humans have yielded conflicting results with regards to improvements in CNS function,^168^ 40% caloric restriction in mice was able to decrease EAE severity and reduce axonal damage.^169^

#### 6.1.2. Ketogenic diet

The ketogenic diet (KD), characterized by high fat, low carbohydrate, and moderate protein intake, induces ketosis, wherein ketone bodies (e.g., *β*-hydroxybutyrate, acetoacetate) become the primary energy source.^170^ KD has shown some success in improving symptoms of neurodegeneration in mouse models of AD and PD.^171^ ^,^ ^172^ Ketone bodies, particularly *β*-hydroxybutyrate (BHB), act as an epigenetic modulator by inhibiting histone deacetylases, leading to increased histone acetylation and suppression of pro-inflammatory microglial activation.^173^ In addition, by blocking K+ efflux, BHB inhibits NLRP3 inflammasome activation in microglia, reducing the release of pro-inflammatory cytokines like IL-1β and TNFα.^174^ Furthermore, in epilepsy, KD stabilizes microglial activity, reducing seizure-induced inflammation and neuronal damage; this is in part due to KD-mediated increases in fiber-fermenting gut bacteria that produce SCFAs to confer anti-inflammatory functions via the gut-brain axis.^175^ Similarly, through BHB and gut microbiota modifications, KD promotes Treg expansion and reduces Th17 cells in mice with EAE, thus decreasing CNS inflammation.^176^

#### 6.1.3. Nutrient supplementation

Nutrient supplementation can address gaps in dietary nutrition and further support CNS function through the mechanisms previously described. Requirements for nutrient supplementation change across the lifespan accordingly with development of the CNS and immune system. For instance, folate is essential for development of the fetal brain and its supplementation during pregnancy prevents neurodevelopmental disorders in offspring.^177^ Recently, taurine supplementation in adulthood has been described to have neuroprotective, cognition-enhancing, and anticonvulsant effects through agonism of glycine and GABA receptors in the CNS.^178^ In old age, omega-3 PUFAs are especially important for preventing neurodegeneration and have shown promise in reducing cognitive decline in early clinical trials of AD patients.

### 6.2. Microbiome-based therapeutics

#### 6.2.1. Probiotics

Probiotic consumption has well-described benefits for gut health, but also has positive effects on CNS immune homeostasis and emerging implications in the treatment of depression,^179^ MS,^180^ and ASD.^181^ In mice, probiotic bacteria (such as *Lactobacillus* and *Bifidobacterium* strains) have been shown to induce intestinal IL-10-producing Tregs and suppress Th1/Th17 cells to dampen intestinal inflammation and strengthen gut barrier integrity,^182^ thus limiting the spread of inflammation to the CNS via the gut-brain axis. In both mice and humans, probiotics have been shown to promote intestinal IgA production,^183^ ^,^ ^184^ which is critical for preventing overgrowth of pathogenic bacteria that produce inflammatory mediators which can traverse the BBB and promote CNS neuroinflammation. Importantly, probiotics may also increase numbers of gut-derived IgA+ B cells in the meninges, where they are believed to participate in steady state CNS immunosurveillance and secrete IL-10 to dampen neuroinflammation in mouse models of MS.^32^ ^,^ ^38^ More recently, the advent of a lactate-producing engineered probiotic was able to suppress EAE symptoms in mice through activation of HIF-1 α signaling in dendritic cells,^185^ highlighting additional strategies for probiotic-dependent modulation of CNS immunity. Numerous clinical trials for probiotic use in the treatment of MS, ASD, AD, and PD are currently ongoing. While these optimal formulations require further investigation, probiotic support of healthy gut microbiota may provide a novel, nutrition-based avenue for modulating CNS immunity and treating brain disorders.

#### 6.2.2. Fecal microbiota transplantation

Fecal microbiota transplantation (FMT) is the most effective method of gut microbiota modulation in humans and is currently approved for the treatment of recurrent *C. difficile* infection. Clinical trials have suggested that healthy donor FMT has the greatest therapeutic potential to decrease symptom severity in ASD patients,^186^ however the mechanistic evidence here remains limited. Several mouse studies have also demonstrated that FMT from healthy mice is effective at correcting gut dysbiosis and alleviating clinical pathology in murine models of PD, MS, AD, and stroke.^186^ These positive outcomes can be ascribed to the increase in beneficial bacteria and metabolites promoting restoration of intestinal and brain barriers, as well as dampening neuroinflammation. Despite these promising preclinical reports, there remains a paucity of clinical trials investigating the application of FMT to treating such neurological disorders in humans. A major challenge here is the still incomplete understanding of what an “ideal” donor gut microbiome that resists neuroinflammation and neurodegeneration is comprised of. Given the emerging role of the gut microbiota in regulating CNS immunity and brain health, there is an imminent need for more mechanistic studies of gut microbiota dysregulation in neurological diseases as well as large randomized controlled trials in human patients to further elucidate the efficacy of FMT in treating neurological disorders.

#### 6.2.3. Concluding remarks

Nutrition plays a pivotal role in modulating immune cell function and microbiota composition to influence CNS health. Early life nutrition, particularly through breast milk components like human milk oligosaccharides, shapes microbial diversity and promotes anti-inflammatory microglia polarization, fostering immune tolerance and optimal neurodevelopment. These early nutritional inputs establish a foundation for CNS resilience, impacting synaptic plasticity and cognitive function. Conversely, aging alters microbiota composition and immune metabolism, driving chronic inflammation and increasing susceptibility to neurodegenerative diseases. These nutritional inputs lay a critical foundation for synaptic plasticity and cognitive resilience. In aging, shifts in microbiota and immune metabolism fuel chronic inflammation, increasing neurodegenerative disease risk. Identifying nutrient-driven immune and neuronal signaling mediators, such as IL-4, IL-17A, or SCFA receptors, offers promising therapeutic targets to enhance neuroimmune fitness. Dietary interventions, including the Mediterranean and ketogenic diets, alongside probiotics, offer promising strategies to enhance neuroimmune fitness by restoring gut barrier integrity and dampening inflammatory signaling. By targeting these mediators, nutritional strategies can optimize neurodevelopment and counteract age-related immune decline, reducing neuroinflammatory and neurodegenerative risks. Future research must prioritize elucidating these signaling pathways and developing precise nutritional interventions to leverage the gut-brain-immune triad for lifelong CNS health and resilience.

Nutritional inputs, such as macronutrient and micronutrient availability, from early life through aging dynamically influence gut microbiota composition and metabolite production. Microbial metabolites such as short-chain fatty acids, tryptophan derivatives, secondary bile acids, and neurotransmitters directly shape intestinal immune cell programming, through various signaling pathways, and trafficking to the CNS. Gut-derived metabolite and cellular signals converge on regulation of CNS cells and essential neuronal functions. The resulting neuroimmune tone shapes optimal brain development and aging, cognitive resilience, and susceptibility to neurological disease. Bidirectional feedback from neuronal and behavioral output returns to modulate nutritional intake and microbial community structure, dynamically maintaining this framework for nutritional regulation of health outcomes throughout the lifespan.

In the intestines, dietary nutrients and metabolites generated by gut bacteria enter the lamina propria and gut-associated lymph nodes to direct immune cell polarization and function. Short-chain fatty acids (SCFAs) promote differentiation of naïve CD4 T cells into both regulatory and effector T cells through activation of cell surface receptors (GPR41, GPR43) and inhibition of histone deacetylase (HDAC). SCFAs also inhibit B cell proliferation, class switching, and differentiation in a HDAC-dependent manner. Retinoic acid binds to retinoic acid receptors (RARα) in naïve T cells to inhibit Th17 cell differentiation, while vitamin D inhibits Th1 cell differentiation through binding to vitamin D receptors (VDR). Retinoic acid has similar anti-inflammatory effects on B cells, inducing the production of IgA + B cells which survey the CNS. Long-chain fatty acids (LCFAs) promote Th1/Th17 differentiation by binding to fatty acid receptors, while bile acid metabolites regulate Th17-Treg balance through direct binding to Foxp3 and RORγt. Intestinal Th1, Th17, and γδ T cells traffic to the CNS and can contribute to neuroinflammation through production of cytokines like IL-17A and IFNγ. Salt and LCFAs promote inflammatory T cell trafficking to the CNS through upregulation of p38/MAPK signaling. Dietary nutrients and secondary metabolites can also cross the blood-brain barrier and directly influence microglia polarization. Omega-3s, SCFAs, and bile acids promote anti-inflammatory microglia polarization by signaling through surface receptors. LCFAs activate TLR4 on microglia and promote proinflammatory functions. Zinc can direct both microglia states in a context-dependent manner. Meningeal γδ T cells and B cells further respond to nutrient-induced changes in the brain microenvironment through cytokine and antibody production. Host nutrition also impacts neuronal signaling and neurotransmitter production by gut bacteria and intestinal enterochromaffin cells (EECs). This leads to stimulation of the vagus nerve which sends afferent signals to the brain and activates an anti-inflammatory pathway that broadly suppresses systemic immunity. In turn, the brain sends efferent signals back to the intestines to regulate gut motility, secretion, and intestinal neurotransmitter production, completing a bidirectional circuit.

**Table 1.** Summary of nutrient effects on different immune cell types within the context of gut-brain signaling and CNS health.

Immune Cell	Nutrient	Effects	Direction of Effect	Source of evidence	Reference
Microglia	Bile acids	Promotes anti-inflammatory microglia and astrocyte polarization through TGR5 and inhibition of NF-κB	Anti-inflammatory	Mice	[64]
	Indole derivatives	Reduces ROS accumulation in microglia and induce production of TGF and VEGF to regulate astrocytes during neuroinflammation	Anti-inflammatory	Mice	[55]
	Human milk oligosaccharides (HMOs)	Reduces ROS accumulation in microglia and promote anti-inflammatory microglia polarization	Anti-inflammatory	Mice	[96]
	Omega-3 polyunsaturated fatty acids	Promotes anti-inflammatory microglia polarization through FFA4 and inhibition of NF-κB	Anti-inflammatory	Mice and human	[21]
	Saturated fatty acids	Promotes proinflammatory microglia polarization through TLR4 and activation of NF-κB; worsens neuron destruction and neuroinflammation	Proinflammatory	Mice	[22]
	Short-chain fatty acids (SCFAs)	Promotes anti-inflammatory microglia polarization through FFAR2/3 and inhibition of NF-κB	Anti-inflammatory	Mice	[187]
	Zinc	Promotes pro/anti-inflammatory microglial polarization in a context-dependent manner	Pro or anti-inflammatory	Immortal murine cell lines	[89,90]
CD4 T cells	Long-chain fatty acids (LCFAs)	Promotes CNS-infiltrating Th1/Th17 cells through p38/MAPK pathway; have been shown to worsen EAE	Proinflammatory	Mice and human	[139]
	Omega-3 polyunsaturated fatty acids	Promotes intestinal Treg differentiation; may have neuroprotective effects in CNS	Anti-inflammatory	Mice	[164,188]
	Retinoic acid	Inhibits IL-6-driven Th17 differentiation, may limit CNS infiltration and neuroinflammation	Anti-inflammatory	Mice	[85,86]
	Salt	Promotes CNS-infiltrating pathogenic Th17 cells through p38/MAPK pathway; high salt diet worsens EAE	Proinflammatory	Mice and human	[145,146,148]
	Bile acid metabolites	Promotes Treg differentiation while inhibiting Th17 by direct binding to RORγt and FOXP3	Anti-inflammatory	Mice	[62]
	Serotonin	Promotes intestinal Treg differentiation by increasing intracellular indole-3-acetaldehyde and inhibiting mTOR activation	Anti-inflammatory	Mice	[68]
	Short-chain fatty acids (SCFAs)	Promotes both effector and regulatory T cell differentiation through histone deacetylase, context-dependent	Pro or anti-inflammatory	Mice	[189]
	Vitamin D	Inhibits Th1/Th17 differentiation and cytokine production by signaling through VDR; supplementation has been shown to ameliorate EAE	Anti-inflammatory	Mice	[88,190,191]
γδ T cells	Retinoic acid	Enhances IL-22 production to promote tissue repair; activation of RAR leads to its binding to *Il22* promoter	Anti-inflammatory	Mice	[192]
	Short-chain fatty acids (SCFAs)	Inhibits IL-17A and IL-22 production through histone deacetylase, potentially dampening intestinal + CNS inflammation	Anti-inflammatory	Mice	[193]
B cells	Retinoic acid	Induces intestinal IgA + B cells which can traffic to the CNS and protect against neuroinflammation	Anti-inflammatory	Mice	[194]
	Short-chain fatty acids (SCFAs)	Inhibits class switch recombination, somatic hypermutation, plasma cell differentiation through downregulation of AID and Blimp1 in a dose-dependent manner; may reduce generation of CNS-autoantibodies	Anti-inflammatory	Mice	[195]
	Vitamin D	Inhibits B cell proliferation, plasma cell differentiation, and IgG secretion; may reduce autoreactive B cell responses	Anti-inflammatory	Mice	[87]
